# Effectiveness of fingolimod in real-world relapsing-remitting multiple sclerosis Italian patients: the GENIUS study

**DOI:** 10.1007/s10072-020-04380-y

**Published:** 2020-04-21

**Authors:** Giancarlo Comi, Carlo Pozzilli, Vincenzo Brescia Morra, Antonio Bertolotto, Francesca Sangalli, Luca Prosperini, Antonio Carotenuto, Pietro Iaffaldano, Marco Capobianco, Delia Colombo, Mihaela Nica, Sara Rizzoli, Maria Trojano

**Affiliations:** 1 San Raffaele Hospital, Milan, Italy; 2 Department of Human Neuroscience, Sapienza University, Rome, Italy; 3 Multiple Sclerosis Center, S. Andrea Hospital, Rome, Italy; 4 Department of Neurosciences, Reproductive and Odontostomatological Sciences, Federico II University, Naples, Italy; 5 A.O.U. San Luigi Gonzaga, Orbassano, Turin, Italy; 6 Department of Neurosciences, S. Camillo-Forlanini Hospital, Rome, Italy; 7 Department of Basic Medical Sciences, Neurosciences and Sense Organs, University of Bari Aldo Moro, Bari, Italy; 8 Novartis Farma S.p.A, Origgio, VA Italy; 9 MediNeos Observational Research, Modena, Italy

**Keywords:** Relapsing-remitting multiple sclerosis, Fingolimod, NEDA-3, Annualized relapse rate

## Abstract

**Background:**

Fingolimod is the first oral agent approved for treatment of relapsing-remitting multiple sclerosis (RRMS). We aimed to evaluate fingolimod effectiveness in a real-world sample of RRMS patients.

**Methods:**

A retrospective, multicentre study in patients treated with fingolimod, whom clinical and radiological data were collected in the 2 years preceding and following the initiation of fingolimod.

**Results:**

Out of 414 patients, 56.8% received prior first-line injectable disease-modifying therapies, 25.4% were previously treated with natalizumab, 1.2% with immunosuppressant agents, and 16.7% were treatment naive. The annualized relapse rate decreased by 65% in the first year and by 70% after two years of treatment. Age ≤ 40 years, ≥ 1 relapse in the 24 months before fingolimod initiation and previous treatment with natalizumab were risk factors for relapses. Overall, 67.9% patients had no evidence of disease activity (NEDA-3) after 1 year and 54.6% after 2 years of treatment. A higher proportion of naïve (81.2% in 1 year and 66.7% after 2 years) or first-line injected patients (70.2% and 56.6%) achieved NEDA-3 than those previously treated with natalizumab (54.3% and 42.9%).

**Conclusions:**

Fingolimod appeared to be effective in naive patients and after first-line treatment failure in reducing risk of relapse and disease activity throughout the 2-year follow-up.

**Electronic supplementary material:**

The online version of this article (10.1007/s10072-020-04380-y) contains supplementary material, which is available to authorized users.

## Introduction

Fingolimod (FNG), proven efficacious in three large phase III trials [1–3] and in their extensions [4, 5], is a widely used oral medication licensed in the USA for the first-line treatment of relapsing-remitting form of multiple sclerosis (RRMS) and in the European Union (EU) for patients with highly active RRMS defined as either high disease activity despite treatment with at least one disease-modifying treatment (DMT) or rapidly evolving severe RRMS. A large number of post-marketing studies have confirmed the efficacy of FNG in a real-world setting, with data substantially overlapping those of pivotal trials [6–13]. All this despite patients are included in observational studies being on average older, with longer disease duration and more severe neurological disability [14]. Moreover, in observational studies, only a minority of patients started FNG as first-line treatment, while the meta-analysis of pooled cohorts of the pivotal FREEDOMS and FREEDOMS II trials extensions showed better outcomes in treatment-naïve patients compared to those previously exposed to other DMTs [15].

So far, most studies have been conducted over short-term follow-up; just a few have reported composite outcomes [8, 10, 16, 17] and have analyzed FNG effectiveness stratified by previous treatment [4, 10, 16–18].

In *FinGolimod Real World EvideNce Italian mUlticenter observational Study in Multiple Sclerosis* (GENIUS study), we conducted a large retrospective real-world data analysis of all the patients referring to five tertiary referral multiple sclerosis (MS) centres in Italy, which initiated FNG during the period 2013–2014 and with a 2-year follow-up, investigating short-term outcomes, namely, annualized relapse rate (ARR) and no evidence of disease activity (NEDA-3) [19], and sustained disability progression. Moreover, in the same patients, a retrospective analysis was carried out up to 2 years prior to the initiation of FNG treatment.

Performing a comprehensive reassessment after 1 and 2 years in a well-characterized group of patients, systematically followed, may provide a unique opportunity to better understand the benefits of FNG on the composed outcomes of disease activity.

## Materials and methods

This study is a secondary data, non-interventional observational cohort study. A retrospective medical chart review method was used to abstract data from hospital medical charts of RRMS patients who initiated FNG treatment within the period 2013–2014. Patients referred to five high-volume tertiary care MS centres, mainly (4 out of 5) academic, located in Italy (in the cities of Turin, Milan, Bari, Rome, Naples).

Data were abstracted from medical charts (paper or electronic) by trained data abstractors and filed in a centralized database for analysis. Data abstractors remained blind to the study hypothesis in order to minimize any kind of bias. A key abstractor leader was named, who trained, assisted, supervised, and audited the site-specific data abstractors. The inter-rater reliability was assessed (see Online Resource #1 for details).

Date of first FNG dispensation was designed as index date. A “pre-FNG period” was defined as the 2 years prior to the index date and the “FNG period” as the 2 years follow-up post index date. Each site abstracted data from about 100 patients’ medical charts, for a planned total number of about 500 patients. Patients inclusion criteria were (a) age ≥ 18 years at index date; (b) diagnosis of MS [20]; (c) starting FNG (> 1 dose) within the period 2013–2014; and (d) available follow-up data for both the pre-FNG and FNG periods. All patients participating in any other clinical trial were excluded. The following endpoints were assessed, both for the pre-FNG period and the FNG period: (a) ARR and (b) NEDA-3 status and its subcomponents (clinical relapses, magnetic resonance imaging (MRI) activity and disability worsening). Relapses were defined as appearance of new neurological deficit that lasted more than 24 h in the absence of fever or infection, which occurred at least 30 days after the onset of a preceding event [21]. Sustained disability progression was defined as a one-point increase sustained for at least 3 or 6 months from an Expanded Disability Status Scale (EDSS) lower or equal to 5.5 or an increase of 0.5 for patients with an EDSS of 6 or greater [22]. The MRI activity was considered in the presence of new T2 lesions (compared to last scan before index date) or Gd-enhancing lesions. Patients with rapidly evolving MS (REMS) were defined as those with two or more disabling relapses in 1 year and evidence of increasing lesions on two consecutive MRI scans.

### Statistical methods

Statistical analyses were carried out both in the entire population and stratified for the last DMT taken by the patient in the pre-FNG period, considering the following groups: (a) patients previously treated with any first-line injectable agents as Betaferon (interferon beta-1b), Rebif (interferon beta-1a), Avonex (interferon beta-1a), Copaxone (glatiramer acetate), and Extavia (interferon beta-1b) (BRACE+); (b) patients previously treated with natalizumab (NTZ+); and c) naïve patients.

FNG treatment discontinuation rates were calculated as the time between first and last administration; patients were censored at drop-out or at the end of observation date.

The annualized change in EDSS was calculated as the difference of EDSS score at FNG start with the one measured after 1- and 2-year follow-up visits multiplied by the actual follow-up period reported to 1 year.

The ARR at 1 and 2 years were calculated by dividing the total number of relapses during 1 and 2 years of treatment by the number of patient-days in the study, and the ratio was multiplied by 365.25. The subject’s time on study was censored at the time of FNG treatment stop (in case of therapy discontinuation) or of the last available visit (in case the treatment was not interrupted). The 95% confidence interval for 1-year and 2-year ARR was also calculated.

Considering the whole sample, two Poisson regression models were estimated in order to evaluate the impact of age at the index date, gender, and number of relapses in pre-FNG period and previous treatment (NTZ+, BRACE+, naïve) on the outcomes (respectively the number of relapses after the first and the second year of FNG treatment). With the help of these statistical models, ARRs (at 1 and 2 years of the FNG treatment period) were estimated in subgroups of patients (i.e., in patients aged ≤ and > 40 years, in patients with 0 and ≥ 1 previous relapses, in men and women, in NTZ+ BRACE+ and treatment-naïve patients), keeping constant the impact of the effects included in the model.

The proportions of patients who had NEDA-3 after 1 and 2 years from FNG start were calculated with their 95% confidence interval. Two multivariate logistic models were developed to estimate the relative risk respectively of (i) being NEDA-3 and of (ii) sustained disability progression during 2 years of FNG treatment period in patients aged ≤ 40 vs > 40 years at FNG start, in men vs women, in patients with no relapses vs ≥ 1 relapses in pre-FNG period, and in BRACE+ and treatment-naïve vs NTZ+ patients.

Site monitoring, data management, and statistical analysis were performed by MediNeos (Modena, Italy). Statistical analysis was performed using SAS v9.4 and Enterprise Guide v7.1.

## Results

### Sample characteristics

We collected data from 522 patients, of which 414 (79.3%) were available for full analysis; 108 patients were excluded from the analysis for the following reasons (multiple reasons admitted): 87 had incomplete/censored records, 42 signed informed consent before approval of the ethics committee, and 2 took only 1 dose of FNG. Patients excluded and available for analysis had similar distribution of sex, age, overall duration of FNG period, previous treatment with natalizumab, evidence of disease activity (according to NEDA-3), MRI disease activity, and EDSS score at FNG start.

Demographic and clinical features of the sample are reported in Table 1.

**Table 1:** Demographic and clinical characteristics at FNG start in the patients with RRMS

	Overall* N = 414	NTZ+ *N* = 105 (25.4%)	BRACE+ *N* = 235 (56.8%)	NAIVE *N* = 69 (16.7%)
Females, *n* (%)	292 (70.5%)	70 (66.7%)	174 (74.0%)	44 (63.8%)
Patient age at FNG start (y), mean (SD)	38.6 (9.7)	39.9 (8.6)	38.1(10.0)	37.9 (10.1)
REMS, *n* (%)	76 (18.4%)	11 (10.5%)	5 (2.1%)	58 (84.1%)
EDSS score at FNG start, median (25°-75°percentile)	2.5 (2.0–4.0)	3.5 (2.0–5.0)	2.5 (1.5–3.5)	2.5 (2.0–3.5)
Patients with ≥ 1 relapse in the 24 months prior to FNG start, *n* (%)	325 (78.5%)	42 (40.0%)	214 (91.1%)	65 (94.2%)
Duration of FNG treatment (months), mean (SD)	22.7 (4.7)	22.4 (4.9)	22.7 (4.7)	22.7 (4.4)

*FNG* fingolimod.

*REMS* rapidly evolving multiple sclerosis

*ARR* annualized relapse rate (considering the 2 years before FNG start)

*BRACE+* patients previously treated with interferon-beta/glatiramer acetate

*NTZ+* patients previously treated with natalizumab

*NAIVE* not previously treated with any disease-modifying treatments

*Overall sample was constituted of 105 NTZ+, 235 BRACE+, 69 NAÏVE, and 5 patients previously treated with immunosuppressant agents. Characteristics of the 5 patients previously treated with immunosuppressants are not reported in the table

The washout period data between last treatment and FNG initiation were not available. Fifty-seven (13.8%) subjects permanently discontinued treatment before the predefined FNG period end: 38 (9.2%) for lack of effectiveness (mean (SD) duration of FNG treatment: 14.1 (6.2) months) and 13 (3.1%) for safety/tolerability issues (mean (SD) duration of FNG treatment: 9.2 (5.8) months).

The median EDSS score was substantially stable over the 2-year follow-up, being 2.5 (25°-75° percentile: 1.5–4.0) at 2 years for the whole sample, 3.5 (25°–75° percentile: 2.0–5.5) for NTZ+, 2.0 (25°-75° percentile: 1.5–3.5) for BRACE+, and 2.5 (25–75° percentile: 2.0–4.0) for treatment-naïve.

### Annualized relapse rate

**Fig. 1:**
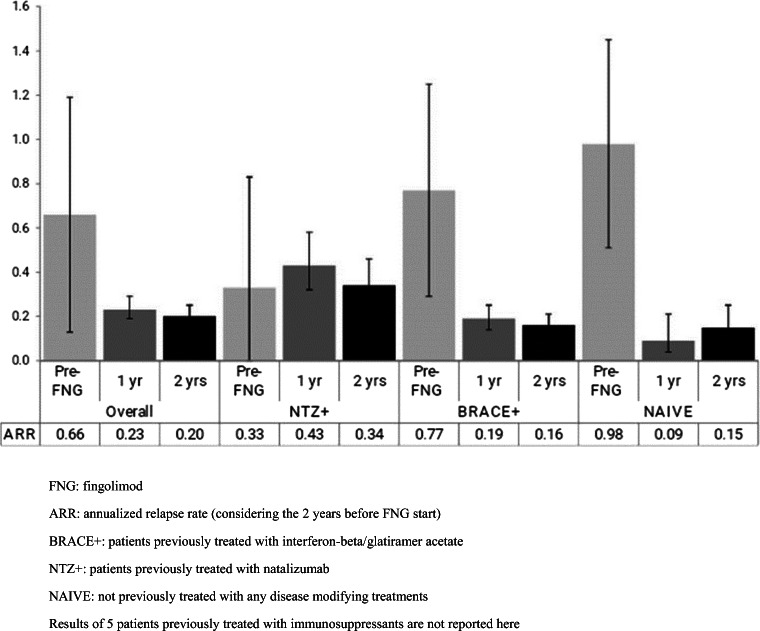
ARR in the pre-FNG period, at 1 year and during the whole FNG period, in the patients with RRMS

Overall, FNG treatment reduced the ARR by 77% in the first year (1-year ARR (95% confidence interval (CI)), 0.23 (0.19–0.29)) and by 80% after 2 years of FNG treatment (2-year ARR (95% CI), 0.20 (0.17–0.25)), as compared to 1-year before index date. FNG treatment reduced the ARR by 65% in the first year and by 70% after two years of treatment (Fig. 1), as compared to the whole pre-FNG period. The ARR analysis stratified by patients’ previous DMT revealed that FNG markedly reduced the ARR in naïve patients by 91% in the first year (0.09; 95% CI, 0.04–0.21) and by 85% after 2 years of treatment (0.15; 95% CI, 0.08–0.25); in BRACE+ by 75% in the first year (0.19; 95% CI: 0.14–0.25) and by 79% after 2 years of treatment (0.16; 95% CI: 0.12–0.21); while in NTZ+, the ARR increased by 30% in the first year (0.43; 95% CI: 0.32–0.58) and by 3% after 2 years of treatment (0.34; 95% CI, 0.25–0.46; Fig*.* 1), as compared to the pre-FNG period.

**Fig. 2:**
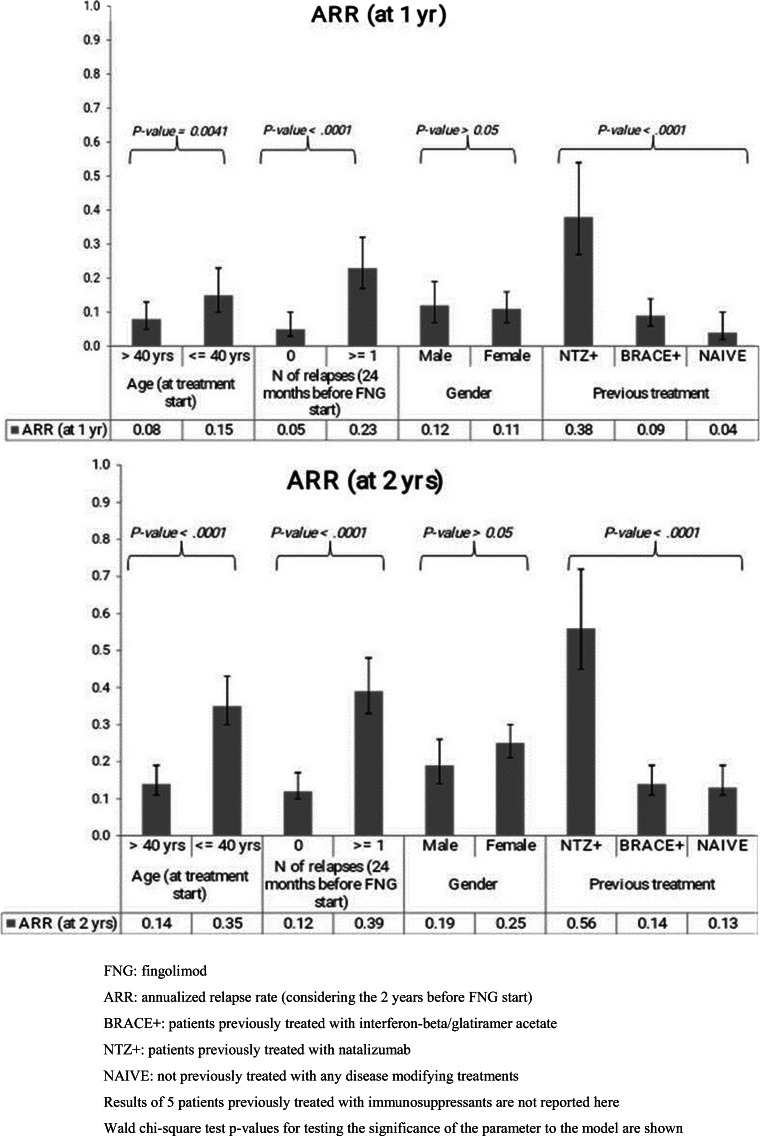
Impact of age, gender, number of relapses (in the 24 months before FNG start), and previous treatment on ARR at 1 year and at 2 years in the patients with RRMS (results of Poisson regression models)

Multivariate Poisson regression revealed that age ≤ 40 years, ≥ 1 relapse in the 24 months before FNG initiation, and previous treatment with NTZ were risk factors associated with relapses under FNG treatment both in the 1 year and over the 2 years of follow-up (Fig. 2). The 2-year ARR was 0.35 (95% CI: 0.26–0.46) in patients aged ≤ 40 years vs 0.14 (95% CI: 0.09–0.20) in patients aged > 40 years, 0.39 (95% CI, 0.31–0.50) in patients with ≥ 1 previous relapse vs 0.12 (95% CI, 0.07–0.19) in patients with no previous relapses and 0.56 (95% CI, 0.43–0.74) in NTZ+ vs 0.14 (95% CI, 0.10–0.20) in BRACE+, and 0.13 (95% CI, 0.07–0.21) in naïve.

### NEDA-3 status

At index date, 79 (19.1%) patients had NEDA-3 in the pre-FNG period: 15 (6.4%) BRACE+, 61 (58.1%) NTZ+ and 3 (4.3%) in the naïve patients group.

In the FNG period, 281 (67.9%, 95% CI: 63.4%–72.4%) patients were NEDA-3 status after 1 year and 226 (54.6%, 95% CI: 49.8–59.4%) after 2 years of treatment. When patients were stratified by previous DMTs, a high proportion achieved NEDA-3 status at follow-up among naïve (*n* = 56, 81.2% (95% CI, 71.9–90.4%) in 1 year and *n* = 46, 66.7% (95% CI, 55.5–77.8%) after 2 years) and BRACE+ patients (*n* = 165, 70.2% (95% CI, 64.4–76.1%) in 1 year and *n* = 133, 56.6% (95% CI, 50.3–62.9%) after 2 years), and in lower rates among NTZ+ patients (*n* = 57, 54.3% (95% CI: 44.8–63.8%) in 1 year and *n* = 45, 42.9% (95% CI, 33.4–52.3%) after 2 years).

Overall, in the FNG period, 83.3% and 75.6% had no relapses, 93.0% and 85.0% patients had no disability progression, and 77.5% and 68.6% patients had no new/enlarged T2 or Gd + lesions, respectively, over the first year and after 2 years of follow-up under FNG treatment (Table 2).

**Table 2:** NEDA-3 subcomponents during the FNG period, in the patients with RRMS

	Overall *N* = 414	BRACE+ *N* = 235	NTZ+ *N* = 105	NAIVE *N* = 69
Relapse-free, n (%)				
1-year FNG period	345 (83.3)	201 (85.5)	77 (73.3)	63 (91.3)
2-year FNG period	313 (75.6)	182 (77.4)	70 (66.7)	57 (82.6)
Lack of MRI activity, n (%)				
1-year FNG period	321 (77.5)	182 (77.4)	74 (70.5)	60 (87.0)
2-year FNG period	284 (68.6)	159 (67.7)	65 (61.9)	55 (79.7)
No sustained disability progression, n (%)				
1-year FNG period	385 (93.0)	222 (94.5)	93 (88.6)	67 (97.1)
2-year FNG period	352 (85.0)	205 (87.2)	84 (80.0)	61 (88.4)

*FNG* fingolimod

*BRACE+* patients previously treated with interferon-beta/glatiramer acetate

*NTZ+* patients previously treated with natalizumab

*NAIVE* not previously treated with NTZ, BRACE or immunosuppressants

NEDA-3 components of 5 patients previously treated with immunosuppressants are not reported here

**Fig. 3:**
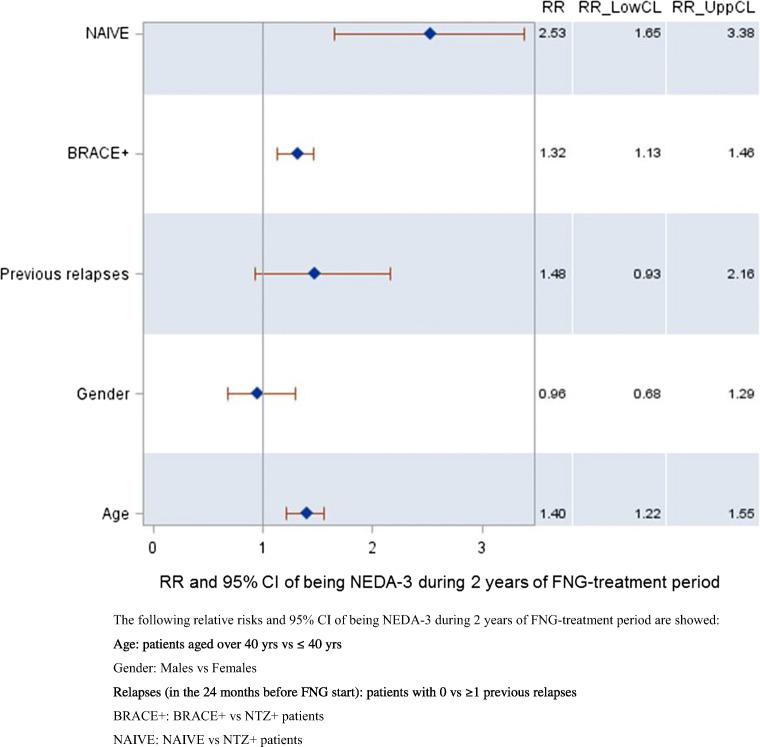
Relative risk of being NEDA-3 during 2 years of FNG treatment period

Multivariate logistic regression analysis on 409 subjects (Fig. 3) showed that being aged > 40 years and not having been previously treated with NTZ significantly increased the risk of keeping NEDA-3 status over 2 years of FNG treatment (kept constant the effect of the variables included in the model). Patients aged > 40 years had 1.40 (95% CI, 1.22–1.55) times the risk of being NEDA-3 in the 2 years than patients aged ≤ 40 years; BRACE+ and naïve patients had 1.32 (95% CI: 1.13–1.46) and 2.53 (95% CI: 1.65–3.38) times the risk of being NEDA-3 compared to NTZ+ patients, respectively.

### Sustained disability progression

**Fig. 4:**
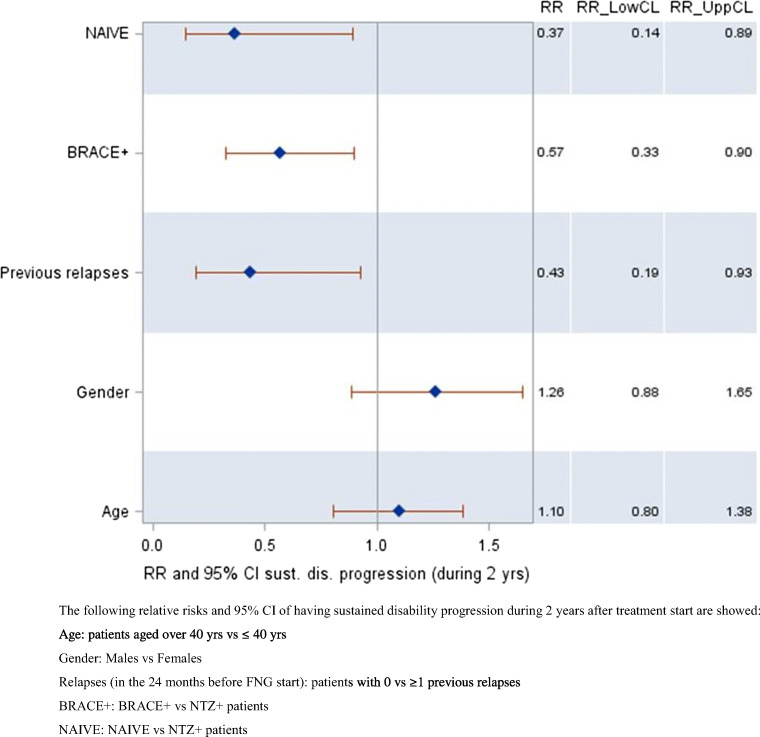
Relative risk of having sustained disability progression during 2 years of FNG treatment period

Multivariate logistic regression analysis on 409 subjects (Fig. 4) showed that, kept constant the effect of the variables included in the model, patients with no relapses in pre-FNG period had about 60% less the risk of having sustained disability progression over 2 years of FNG treatment than patients with ≥ 1 relapses during the pre-FNG period (RR, 0.43; 95% CI, 0.19–0.93); BRACE+ and treatment-naïve patients had, respectively, about 40% (RR, 0.57; 95% CI, 0.33–0.90) and 60% (RR: 0.37; 95% CI: 0.14–0.89) less the risk of having sustained disability progression over 2 years compared to NTZ+ patients. Age and gender had no effect on the risk for sustained disability progression over 2 years of FNG treatment.

## Discussion

In MS, there is a growing emphasis on obtaining data of “real” patients, beyond randomized clinical trials. Post-marketing studies are important for providing information on compliance with current treatment guidelines, identifying suboptimal therapies, defining treatment responder subgroups, optimizing treatment algorithm, and detecting rare serious adverse events. Although not providing a level of evidence as high as randomized controlled trials, real-world studies provide crucial information on effectiveness outcomes of a given therapy, by investigating a more diverse group of patients than those included in clinical trials. Real-world studies provide data that can be generalized across the population of patients with MS in clinical practice.

The GENIUS study used clinical and MRI data collected in clinical practice to assess FNG effectiveness in terms of ARR, NEDA-3 status, and disability progression in a large cohort of patients with RRMS in Italy. Real-world FNG effectiveness data were analyzed, stratifying patients into treatment-naïve ones and those previously treated with other DMTs.

Our results largely confirm the effectiveness data of FNG in reducing ARR in RRMS, by 65% and 70% reduction in risk of relapses, in the 1-year and 2-year of treatment, respectively. Notably, in naive subjects, the ARR reduction is more marked than in patients starting FNG after other DMTs. In NTZ+, while observing a slight increase in ARR in the first year, a substantial stabilization is achieved by the second year of treatment.

The effectiveness results obtained in our study are in line with the efficacy data shown in the 0.5-mg/day FNG arm of the 12-month TRANSFORMS and the 24-month FREEDOMS trial [1–3], despite our sample was composed of a lower percentage of naïve patients, who generally show a greater response to FNG, and of a larger rate of NTZ+ patients, who instead are more at risk of disease reactivation [4, 13, 23, 24]. Regarding the NEDA-3 status, our study showed that 67.9% and 54.6% of RRMS patients was NEDA-3 after the first year and the second year of treatment. Our results are similar to those obtained in other cohorts of patients. Prosperini et al. showed that in patients switching from other treatments, FNG delivered a higher proportion of NEDA-3 patients than dimethyl fumarate, after 18 months of follow-up, while the two treatments had similar results in naive patients [16]. Similarly, in another Italian cohort, almost half of patients maintained NEDA-3 status after 2 years of FNG treatment, with rates comparable to ours both in NTZ+ patients and in patients previously treated with other DMTs [8].

To our knowledge, this is one of the largest real-world series of patients with RRMS treated with FNG with composite outcomes assessment and for which composite effectiveness outcomes assessment data are available for the 2 years before and after FNG treatment initiation. Similarly to other real-world studies [6, 7, 11, 12, 24], patients under FNG treatment maintained a low ARR and thus a high proportion of relapse-free patients, as compared to the pre-FNG period, with a high rate of treatment persistence. Regarding the subgroup analysis, our data showed that FNG brings a benefit on MS patients regardless of the previous DMT used, although to a lesser extent in the NTZ+. Although NTZ+ patients showed an increased ARR in the first year of treatment with FNG, two thirds of them were relapse-free after 2 years, more than half of them maintain NEDA-3 status in the first year and 42% in the second year of treatment. These results are in line with the previous observations [6, 11, 13, 18, 24–26]. Furthermore, we confirmed that previous NTZ treatment, just as younger age, and ≥ 1 relapse in the pre-treatment year are significantly associated with an increased risk of relapsing under FNG treatment, as previously demonstrated [8]. The risk of having sustained disability progression is also influenced by the relapses in the pre-treatment period (≥ 1 relapse) and the previous NTZ treatment but not by the younger age. We did not collect data about the duration of the washout period between NTZ cessation and the start of FNG. This prevents us drawing any definite conclusions, as timing of treatment initiation may be critical for achieving an optimal effect [27, 28].

The GENIUS study has several strengths. First, it is the largest real-word FNG study conducted in Italy so far, and it provided evidences for effectiveness outcomes referred to a short-term observation period. Second, homogeneous data collection and centralized data analysis ensured results reliability. Third is the use of NEDA-3 as a composite outcome for effectiveness evaluation, in addition to the ARR. Finally, the subgroup analysis based on previous treatments is a valuable data to a better definition of therapeutic algorithms.

The limits of the study are intrinsic to the retrospective nature of the analysis. Our study has collected data from five different tertiary referral MS centres in Italy of which 4 were academic. Although outcomes were assessed in a large cohort, we cannot exclude a selection bias and a certain lack of homogeneity in the clinical assessments; however, as recently demonstrated by an American real-world study [17], no significant differences exist between academic and non-academic centres in proportion of patients who achieved NEDA-3 at 24 months after FNG. Moreover, in our study, the reading of MRI scans was not centralized, and detailed data on tolerability and safety (in terms of occurred adverse events) have not been collected; in any case, the discontinuation rates for safety and tolerability issues are in line with clinical and observational studies. Finally, in the absence of a comparison group, our findings should be interpreted with caution since longitudinal data may be influenced by the regression to the mean phenomenon [29].

## Conclusions

FNG provided consistent effectiveness benefits in the Italian real-world setting. This was observed also after treatment with other DMTs across a range of subgroups of patients with relapsing MS. The magnitude of the beneficial effect of FNG may depend on age and type of previous treatment. These findings suggest that most benefit will be gained by patients who start FNG early in the disease course, but the findings also suggest that FNG treatment will benefit patients later in the disease course when they have already accrued disability.

## Electronic supplementary material

ESM 1 (DOCX 23 kb)
