# Supplementary material for: Effectiveness of fingolimod in real-world relapsing-remitting multiple sclerosis Italian patients: the GENIUS study
Source: Neurol Sci. 2020 Apr 21;41(10):2843–51. doi: 10.1007/s10072-020-04380-y (PMC7479005; doi:10.1007/s10072-020-04380-y)
Supplement: Supplementary file 1 — (DOCX 23 kb) [file 10072_2020_4380_MOESM1_ESM.docx]

### Supplementary Material #1

INTER-RATER RELIABILITY

Research studies that involve abstraction of patients’ medical records rely on the assumption that necessary data will be present in the record, in a form adequate for abstraction, accurate and consistent throughout the record, and interpreted in a consistent manner by all the abstractors involved in the study.

Data abstractors of the GENIUS study were carefully selected and trained [Allison et al. 2000; Pan et al. 2005; Wu and Ashton 1997]; training included a careful review of the variables, the procedural manual, and the data abstraction form. No more than two data abstractors for each site were used for data abstraction during the study, in order to conduct operations of data collection and to provide a reliability testing.

Within GENIUS study a Key Abstractor Lead was named across all sites, who trained, assisted, supervised and audited site-specific data abstractors. Data abstractors remained blind to the study hypothesis to minimize “subjectivity in classification in relation to personal theories about the study’s aims”.

In order to collect data, data abstractors reviewed and coded each chart, playing an important role with respect to data quality. Coding must be performed accurately and consistently, or the validity of the data might be compromised.

Literature warns about the potential limits of misinterpretations during abstraction. As a consequence inter-rater reliability (IRR) was evaluated in the GENIUS study.

Inter-rater reliability is a calculated statistical estimate that reports if data are consistent between raters; it specifically measures the ability of two or more independent abstractors to reproduce identical coding. Inter-rater reliability may also be thought of as a measure of the amount of error among the coders of the data variable set [Worster and Haines 2004]. The Cohen’s K and the rate of discrepancy among the coders were calculated. A calculation of percent agreement only indicated the agreement of coders within similar or identical abstractions, whereas Cohen’s k evaluated the extent of agreement between/among coders compared to the total agreement possible while restricting for the possibility of agreement by chance [Worster and Haines 2004].

Magnitude guidelines have appeared in the literature for Cohen’s k. Landis and Koch [[Landis and Koch 1977]](https://en.wikipedia.org/wiki/Cohen%27s_kappa#cite_note-LandisKoch1977-12) characterized values < 0 as indicating no agreement and 0–0.20 as slight, 0.21–0.40 as fair, 0.41–0.60 as moderate, 0.61–0.80 as substantial, and 0.81–1 as almost perfect agreement. This set of guidelines is however by no means universally accepted. Fleiss's [[Fleiss 1981]](https://en.wikipedia.org/wiki/Cohen%27s_kappa#cite_note-Fleiss1981-14) equally arbitrary guidelines characterize kappas over 0.75 as excellent, 0.40 to 0.75 as fair to good, and below 0.40 as poor.

Before starting the study we were not aware of which kind of variables were subject to possible different interpretations. Therefore a step-by-step process was followed, consisting of the following evaluations:

1. Pre-assessment (performed by the Lead abstractor on a random sample of 10% of abstracted charts) with the aim to identify forms and variables subject to the highest variability in interpretations. Cohen’s K and the rate of discrepancy by form were calculated. At the end of pre-assessment the form of DISEASE ACTIVITY (collecting data about number of relapses, EDA, MRI disease activity, sustained disability progression and EDSS score) was identified as the most tricky to be filled in. For this reason, the inter-rater reliability (IRR) test was performed on this form.
2. IRR testing detailed as follows:
   1. Random extraction of patients to be tested (10%)
   2. Data extraction and input of patients on a different environment by the 2° rater
   3. Comparison between raters. According to Fleiss's guidelines [Fleiss 1981] Cohen’s Kappa was excellent for all fields of the DISEASE ACTIVITY form.
   4. Reconciliation of discrepancies (Raters + lead abstractor). No changes were applied to clinical data base because investigators from all sites confirmed the data in the clinical data base.

After the IRR test and the reconciliation phase the quality of the data base was considered reliable and appropriate for data analysis.
